# Non-verbal Intelligence in Unilateral Perinatal Stroke Patients With and Without Epilepsies

**DOI:** 10.3389/fped.2021.660096

**Published:** 2021-05-31

**Authors:** Alisa Gschaidmeier, Magdalena Heimgärtner, Lukas Schnaufer, Pablo Hernáiz Driever, Marko Wilke, Karen Lidzba, Martin Staudt

**Affiliations:** ^1^Department of Pediatric Neurology and Developmental Medicine, University Children's Hospital, Tübingen, Germany; ^2^Center for Pediatric Neurology, Neurorehabilitation and Epileptology, Schön Klinik Vogtareuth, Vogtareuth, Germany; ^3^Experimental Pediatric Neuroimaging, Children's Hospital and Department of Neuroradiology, University Hospital, Tübingen, Germany; ^4^Department of Pediatric Oncology and Hematology, Berlin Institute of Health, Charité-Universitätsmedizin Berlin, Freie Universität Berlin, Humboldt-Universität zu Berlin, Berlin, Germany; ^5^Division of Neuropediatrics, Development, and Rehabilitation, University Children's Hospital Inselspital, Bern University Hospital, University of Bern, Bern, Switzerland

**Keywords:** early brain lesion, functional magnet resonance imaging, lesion size, motor impairment, cognitive function

## Abstract

**Background:** The risk factors for impaired cognitive development after unilateral perinatal stroke are poorly understood. Non-verbal intelligence seems to be at particular risk, since language can shift to the right hemisphere and may thereby reduce the capacity of the right hemisphere for its originary functions. Pharmaco-refractory epilepsies, a frequent complication of perinatal strokes, often lead to impaired intelligence. Yet, the role of well-controlled epilepsies is less well-understood. Here, we investigated whether well-controlled epilepsies, motor impairment, lesion size, lesion side, and lateralization of language functions influence non-verbal functions.

**Methods:** We recruited 8 patients with well-controlled epilepsies (9–26 years), 15 patients without epilepsies (8–23 years), and 23 healthy controls (8–27 years). All underwent the Test of Non-verbal Intelligence, a motor-independent test, which excludes biased results due to motor impairment. Language lateralization was determined with functional MRI, lesion size with MRI-based volumetry, and hand motor impairment with the Jebson-Taylor Hand Function-Test.

**Results:** Patients with epilepsies showed significantly impaired non-verbal intelligence [Md = 89.5, interquartile range (IQR) = 13.5] compared with controls (Md = 103, IQR = 17). In contrast, patients without epilepsies (Md = 97, IQR = 15.0) performed within the range of typically developing children. A multiple regression analysis revealed only epilepsy as a significant risk factor for impaired non-verbal functions.

**Conclusion:** In patients with unilateral perinatal strokes without epilepsies, the neuroplastic potential of one healthy hemisphere is able to support the development of normal non-verbal cognitive abilities, regardless of lesion size, lesion side, or language lateralization. In contrast, epilepsy substantially reduces this neuroplastic potential; even seizure-free patients exhibit below-average non-verbal cognitive functions.

## Introduction

Perinatal stroke has an estimated birth-prevalence of 37–64/100,000 (1, 2). It affects mostly term-born newborns, and presents with diverse signs and symptoms (2). Outcome is quite variable, with many children achieving normal levels of function (3, 4), while others experience difficulties, such as impairment in different domains of cognitive functions (5–7) as well as in motor skills (8–10). Potentially modifying factors explaining the variability in cognitive outcome are lesion size, the severity of hand motor impairment (6), and the side of the lesion (11–13).

In addition, for non-verbal functions in patients with left-sided lesions, language reorganization has been proposed as a modifying factor (6, 7, 14). This has been explained in the context of the “crowding hypothesis” (15–17), suggesting that cognitive processes originally located in the right hemisphere such as non-verbal intelligence show deficits when language functions shift to the right hemisphere. Following this hypothesis, non-verbal functions seem to be at a particular risk for lower performance; hence, in this study, we chose to focus on the development of non-verbal functions after perinatal stroke.

The threat most feared by families of children with perinatal stroke, however, is the development of epilepsy. Indeed, patients with perinatal stroke have a significant risk to develop epilepsy, which was estimated between 15% (18) and 54% (19). Not surprisingly, the type of the lesion also plays an important role, since children with cortico-subcortical lesions (commonly due to arterial ischemic strokes, AIS), are much more prone to develop epilepsies than children with white matter lesions (usually due to periventricular venous infarctions, PVI) (7, 20). In addition to the burden of seizures and side effects of anti-epileptic medication, epilepsy may also hamper cognitive development. For children with pharmaco-refractory epilepsies due to perinatal strokes, it is well-known that cognitive development can be severely compromised (3, 4, 21, 22). Much less is known, whether less severe, well-controlled epilepsies also play a role in this regard.

A frequent methodological problem in almost all previous studies on non-verbal cognitive abilities after perinatal stroke was the application of tests requiring bimanual activities, like the Wechsler Intelligence Scales (3, 4, 23–25), the Kaufman Assessment Battery for Children (7), or the Beery Developmental Test of Visual-Motor Integration (4). Administering such tests in hemiparetic patients—a frequent consequence of perinatal stroke (9, 10)—can lead to artificially lower scores for non-verbal functions [see (26) for review]. To overcome this problem, some authors [e.g., (6)] simply excluded patients with substantial motor impairments that prevented valid administration of the measures. As this potentially biases the outcomes, we here opted to use a different approach: the assessment of non-verbal cognitive abilities with completely motor-free tests. In a previous study (14), we had used the Block Tapping test and the Tube Figures Test; in the current study, we used the Test Of Non-verbal Intelligence (TONI-4) which measures the ability for abstract reasoning and the problem-solving capability—without involving any motor component (27, 28).

Motor impairment may therefore influence the result of cognitive tests by different mechanism. First, as described above, motor impairment can artificially influence the test procedure itself. Second, impaired motor abilities might limit a child's abilities to explore its environment, and thereby impair also the development of cognitive functions (29).

The aim of our study therefore was to assess the influence of well-controlled epilepsy (which we arbitrarily defined as seizure-freedom for at least 6 months) on non-verbal cognitive development of children with perinatal strokes, as assessed using appropriate test procedures. Furthermore, we wanted to clarify the influence of lesion size, hand motor impairment, side of the lesion, and language lateralization on non-verbal cognitive performances.

## Method

### Subjects

Participants were recruited in the University Children's Hospital Tübingen and in the Center for Pediatric Neurology, Neurorehabilitation and Epileptology, Schön Klinik Vogtareuth.

In Tübingen, participants were recruited by searching the clinical database for relevant diagnoses in electronic patient charts and by personal contacts to patients who had participated in previous studies. Healthy controls were recruited *via* advertisements in the local press and in the clinic internal information system. Patients in Vogtareuth were recruited *via* personal invitations after searching the clinical database and during hospitalization for a motor skills rehabilitation training. These differences in recruitment strategies may explain why the two cohorts of patients differed in terms of age (median age Tübingen = 18.13 years, median age Vogtareuth = 10.15 years). Inclusion criteria, however, were the same, and all participants were included following telephone interviews with identical questionnaires.

We included 23 patients (11 females; age range 8–26 years; median age 12.56 years) with a diagnosis of a pre-, peri-, or neonatally acquired unilateral arterial ischemic stroke (AIS) or unilateral periventricular hemorrhagic infarction (PVI). In order to be able to participate in all tests, patients had to be native German-speaking and aged 8 years or older. Patients with a previous diagnosis of intellectual disability (defined as IQ below 70) were excluded. Controls were screened using a questionnaire asking for any neurological or psychiatric diagnosis and for problems in cognitive or language development. We did not, however, use any formal assessment confirming normal development.

Additionally, patients with seizures during the last 6 months were excluded. Hemiparesis was present in 21/23 patients (no hemiparesis in #V13, #T12).

Patients were diagnosed with epilepsy (*n* = 8; age range, 9–26 years; median age, 16.40; 2 females) when at least two afebrile, unprovoked seizures had occurred in the post-neonatal period [definition as suggested in (19, 30)]. All but one of these patients (#T28) were on anti-convulsive medication at the time of the study. Of the patients without epilepsies (*n* = 15; age range, 8–23 years; median age, 11.80; 9 females), one patient (#V03) had suffered one single seizure, but was never treated with anti-convulsive medication. The other 14 patients never had experienced epileptic seizures. Twenty-three age-matched healthy volunteers (age range 8–27 years; median age, 12.42 years; 8 females) served as controls. For every single patient, we age-matched a person from our cohort of 38 controls—irrespective of epilepsy.

The study was approved by the local ethics committee (Nr. 693/2014B01). All adult participants and the parents of underage participants gave their written, informed consent, and all underage participants gave verbal assent. The study was in accordance with the Code of Ethics of the World Medical Association (Declaration of Helsinki, 1964 in its latest version).

### Neuropsychological Assessment

All participants completed the Test of Non-verbal Intelligence, Fourth Edition (TONI-4), which measures the ability for abstract reasoning and the problem-solving capability (28). It contains of 60 graphical items, arranged in order from easy to difficult. The participants analyze similarities and differences between the items, which are defined by the characteristics shape, position, direction, rotation, contiguity, shading, size and movement. The age-adjusted test results are not confounded by motor deficits and are therefore suitable for patients with motor impairments such as hemiparesis (27).

### Structural and Functional Magnetic Resonance Imaging

Subjects were either scanned on a Siemens 1.5 T Avanto (Tübingen) or Symphony (Vogtareuth) MRI scanner (Siemens Medizintechnik, Erlangen, Germany), using the same sequences and a standard quadrature head coil. An echo-planar imaging (EPI) sequence was used to acquire functional series (repetition time = 3,000 ms, echo time = 40 ms, 40 axial slices, in plane matrix = 64 × 64, covering the whole brain with a voxel size = 3 × 3 × 3 mm3). Anatomical images were acquired as T1-weighted 3D-datasets (repetition time = 1,300 ms, echo time = 2.92 ms, 167 contiguous sagittal slices, in plane matrix 265 × 265, resulting in a voxel size of 1 × 1 × 1 mm3).

Functional and anatomical images were pre-processed and analyzed using SPM12 (Statistical Parametric Mapping; Wellcome Department of Imaging Neurosciences, UCL, UK), the Computational Anatomy Toolbox extension to SPM12 (CAT12, by Christian Gaser and Robert Dahnke, Departments of Psychiatry and Neurology, Jena University Hospital), as well as custom scripts and functions running within Matlab (Mathworks, Natrick MA, USA).

For the determination of lesion size, individual lesion masks were manually drawn in native space on the anatomical T1-weighted image using MRIcron (31). To compensate for asymmetric ventricular enlargement, the mirrored ventricles of the contra-lesional hemisphere were excluded from the lesion masks for all subjects. Lesion volumes were calculated from the lesion masks using a custom script.

For the determination of language lateralization, all patients with left-sided lesions underwent fMRI and performed the *Vowel Identification Task* (32) as a word generation task. Methodological details have been described elsewhere (32–34). In brief, pictures of everyday objects were presented visually to the participants, who were asked to decide if the name of the object contained the phoneme < i> by silently generating the name of the object. In the control condition, participants were presented an abstract puzzle and were asked to decide if a small piece fitted into a larger one. Laterality of language activation was calculated as described in Lidzba et al. (35). Based on the resulting laterality index (LI), patients were classified as “left-dominant” (LI > +0.2), as “right-dominant” (LI < −0.2) or as “bilateral” (−0.2 ≤ LI ≤ +0.2).

### Motor Assessment

Hand motor function was assessed with the Jebson Taylor Hand function test (JTHFT). The test provides quantitative measurements of standardized unimanual hand function tasks that are frequently used in everyday activities (36). We calculated the median of six subtests (card turning, picking up small objects, stacking checkers, simulated feeding, lifting light objects and lifting heavy objects) (37). As suggested previously, we did not perform the subtest “writing” due to possible distortion by the different ages and performance levels of the participants (38–40). The test was initially developed for adults, but recently showed a good test-retest reliability in children aged 6–10 years (41). For our analysis, we calculated the ratio of the medians “non-dominant”/“dominant” hand motor function to control for inter-individual differences not related to the hemiparesis.

### Statistics

The statistical analyzes were performed using SPSS 25. For correlation analyses, we used Spearman rank correlations; for correlations including dichotomous variables, we used the point-biserial correlations. Significance was assumed at *p* ≤ 0.05, For group comparisons between the three groups, we used the non-parametric Kruskal-Wallis-test, corrected for multiple comparisons where appropriate by Bonferroni correction. A Chi-Square-test was used for a distribution measurement of the control group. Multiple regression analyses were used for the assessment of several predictor variables for the outcome variable non-verbal intelligence.

## Results

Table 1 summarizes demographic and clinical data of all 23 patients. Lesion size could be calculated in 21/23 patients, ranging from 1.16 to 220.71 cm3. In the other two, dental braces (#V04) or an implanted shunt system (#T28) prevented the reliable determination of lesion size (Table 1). The presence of epilepsy correlated significantly with lesion size (point-biserial correlation, *r* = −0.47, *p* < 0.01) and with the type of the lesion (AIS vs. PVI, Phi *p* < 0.05).

**Table 1:** Patient characteristics.

**ID**	**Age at study (years)**	**Sex**	**Lesion type**	**TONI**	**Epilepsy**	**Lesion size (ml)**	**JTHFT (ratio)**	**Lesion side**	**LI**	**Age of onset (years)**	**Time since seizure-freedom (years)**	**History of status epilepticus**	**History of AEDs**	**Time between first and last seizure (years)**
T14	16	M	AIS	77	Yes	133.93	1.37	Left	−0.5	11	1.25	No	LEV BRIV, OXC	4
T39	26	M	AIS	87	Yes	167.79	25.34	Left	−0.87	11.5	8	No	STM	1.5
T44	16	M	AIS	87	Yes	84.97	9.48	Right		N/A	N/A	N/A	OXC	
T41	22	M	PVI	89	Yes	200.57	6.49	Right		15.5	6	No	OXC	1
V11	10	M	PVI	90	No	50.29	4.69	Left	+0.63					
T28	16	M	Not classified^*^	90	Yes	N/A	1.52	Left	+0.69	4	1	Yes	STM, LEV, CLB, PHT	12
V04	15	F	PVI	91	No	N/A	5.75	Right						
T21	23	F	PVI	92	No	29.57	6.64	Left	−0.25					
V14	10	M	PVI	93	No	79.38	18.65	Right						
V06	8	M	PVI	93	No	28.83	2.40	Left	+0.78					
T57	19	F	PVI	93	No	1.16	1.97	Left	+0.64					
T33	23	M	PVI	97	No	161.77	4.08	Left	−0.66					
V03	11	F	PVI	97	No	55.82	2.94	Left	+0.67					
T19	23	F	PVI	99	No	36.05	2.42	Left	−0.83					
T13	9	M	PVI	99	Yes	22.82	1.58	Left	+0.85	0.25	0.5	Yes	LEV, ACTH, PB, STM, CLB, Steroids, ESM	8
V01	8	M	PVI	100	No	24.80	15.25	Left	+0.61					
V12	10	F	PVI	101	Yes	220.71	5.05	Right		1	3	No	OXC, TPM, LEV	6
V13	10	F	PVI	102	No	85.36	0.95	Left	−0.31					
V02	8	F	PVI	104	Yes	45.11	2.04	Right		4.5	3	No	LEV	1.5
V09	10	F	PVI	108	No	1.24	2.17	Left	N/A					
T12	12	F	PVI	109	No	97.30	1.12	Left	−0.32					
T56	12	M	PVI	109	No	9.08	2.39	Right						
V08	11	F	AIS	121	No	41.65	3.85	Left	−0.53					

*Patients are sorted by TONI. Hand motor function is calculated by the ratio (non-dominant/dominant) hand function of the median scores of JTHFT*.

*AIS, arterial ischemic stroke; LI, laterality index (positive: left; negative: right); N/A, no data available due to incompliance or technical failure; PVI, periventricular venous infarction; JTHFT, Jebson Taylor Hand function test; TONI, Test of non-verbal intelligence. LEV, Levetiracetam; BRV, Brivaracetam; OXC, Oxcarbazepine; STM, Sulthiame; CLB, Clobazam; PHT, Phenytoin; PB, Phenobarbital; ESM, Ethosuximide; TPM, Topiramate*.

* *The lesion in T28 could not unequivocally be classified as AIS or PVI because only MRI after implantation of a ventriculoperitoneal shunt were available*.

**Figure 1:**
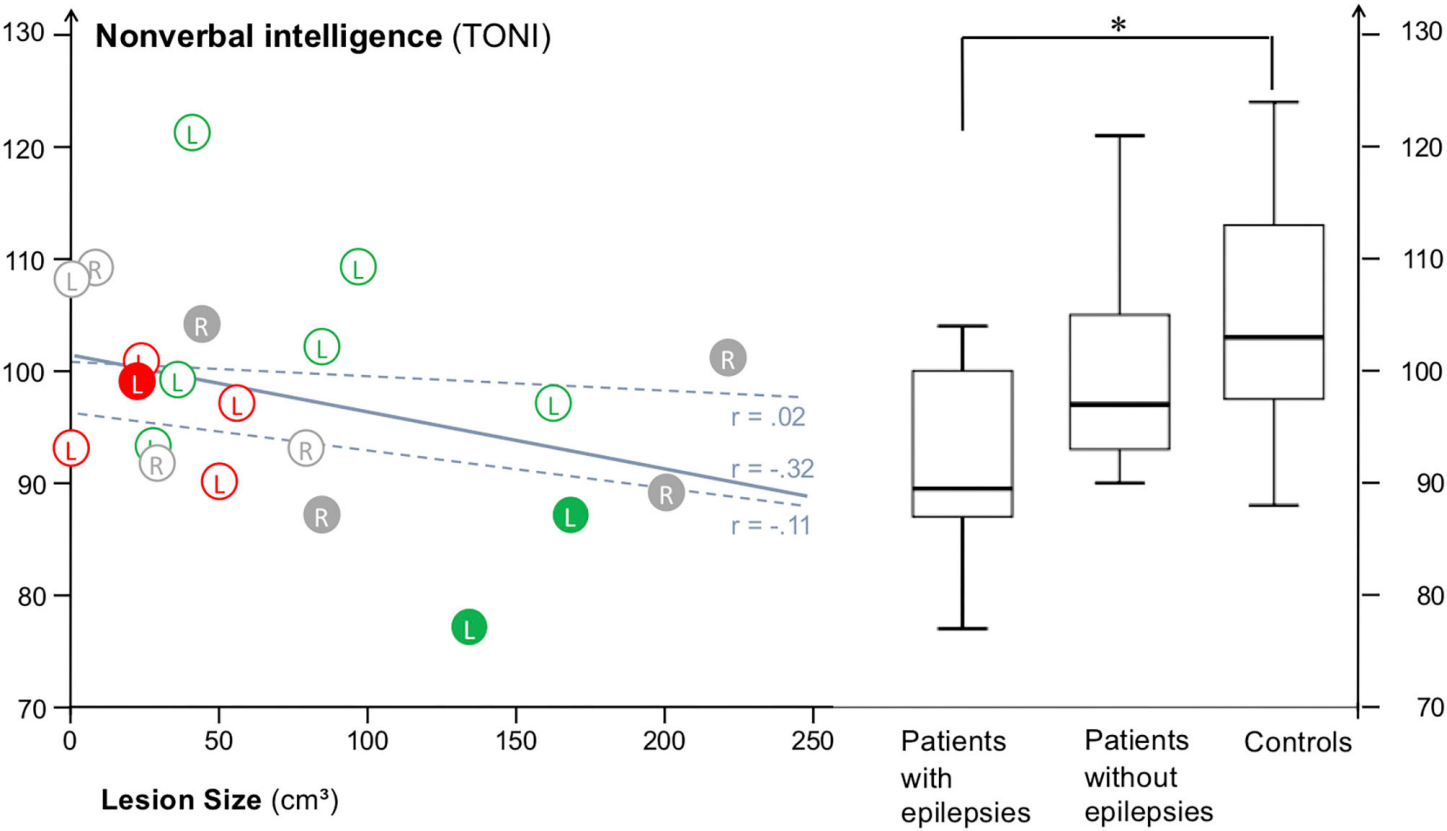
Scatterplot of all 21 patients with lesion size data available, marked with solid circles (with epilepsy) or open circles (without epilepsy). Capital letters mark the side of the lesion (L, R), colors mark the lateralization of language (red = left-dominant; green = right-dominant; gray = no information). The solid line indicates the correlation between lesion size and TONI for all patients, the dashed lines the correlations for the subgroups of patients with epilepsies (lower line) and without epilepsies (upper line). The median TONI scores were about 7.5 points lower for patients with epilepsies (Md = 89.5, SD = 9.0) vs. those without epilepsies (Md = 97, SD = 8.8), and almost one standard deviation lower for patients with epilepsy vs. typically developing controls (Md = 103, SD = 10.0). Patients with epilepsy differed significantly (Kruskal-Wallis-test) from controls (marked with *), while patients with and without epilepsy and patients without epilepsy and controls did not differ.

The Kruskal Wallis-test for the three groups—patients with epilepsy, patients without epilepsy, and controls—yielded a significant main effect for non-verbal intelligence [H (2) = 9.06, *n* = 46, *p* < 0.05]. Pairwise comparisons with Bonferroni-adjusted *p*-values demonstrated that patients with epilepsy scored significantly lower than controls (*p* = 0.010), while no significant differences were observed between patients without epilepsy and controls (*p* = 0.364) and between patients with and without epilepsy (*p* = 0.341; Figure 1).

Results of the assessment of potential modifiers of non-verbal intelligence are displayed in Table 2. TONI scores were negatively correlated with the presence of epilepsy (point-biserial correlation, *r* = −0.41, *p* < 0.05) and with motor impairment of the paretic hand (Spearman Rank, *r* = −0.36, *p* < 0.05). Lesion size showed a trend (*r* = −0.32; *p* = 0.078) toward inferior non-verbal intelligence scores in patients with larger lesions. No significant correlations were observed for lesion side (*r* = 0.04; *p* = 0.42) nor, in patients with left-sided lesions, for language lateralization (*r* = 0.08; *p* = 0.40).

**Table 2:** Correlation coefficients.

	**Epilepsy**	**JTHFT (ratio)**	**Lesion size**	**Lesion side**	**LI (left-sided lesions only)**
TONI-4	−0.405*	−0.359*	−0.324	−0.042	−0.075

*Correlation coefficients between non-verbal intelligence (TONI), hand motor impairment (JTHFT-ratio), lesion size, lesion side, and language lateralization (Spearman rank, one-tailed; for lesion side as a dichotomous variable: point-biserial correlation, one-tailed). Note that lesion size was available for only 21/23 patients, and only 15/16 patients with left-sided lesions contributed data for language lateralization*.

The bold values with *indicate a statistically significant correlation.

To investigate the differential effects of the potential modifiers epilepsy, hand motor impairment, lesion size, and lesion type, we conducted a stepwise linear multiple regression analysis in the order of the strength of the correlation coefficients. Only the factor epilepsy (*R*^2^ = 0.17, beta = −0.410, *t* = −2.0, *p* < 0.05, one-tailed) was retained in the model as a statistically significant predictor, with epilepsy explaining 17 % of the variance in non-verbal intelligence. Neither hand motor impairment (*p* = −0.13, one-tailed) nor lesion size (*p* = 0.34, one-tailed) provided additional information.

Non-verbal intelligence did not correlate with epilepsy onset (*r* = −0.61, *p* = 0.15), time since seizure freedom (*r* = 0.20, *p* = 0.67), number of different antiepileptic drugs ever used (*r* = 0.19; *p* = 0.65), time between first and last seizure (*r* = 0.20, *p* = 0.67) or history of status epilepticus (*r* = −0.10, *p* = 0.85).

**Figure 2:**
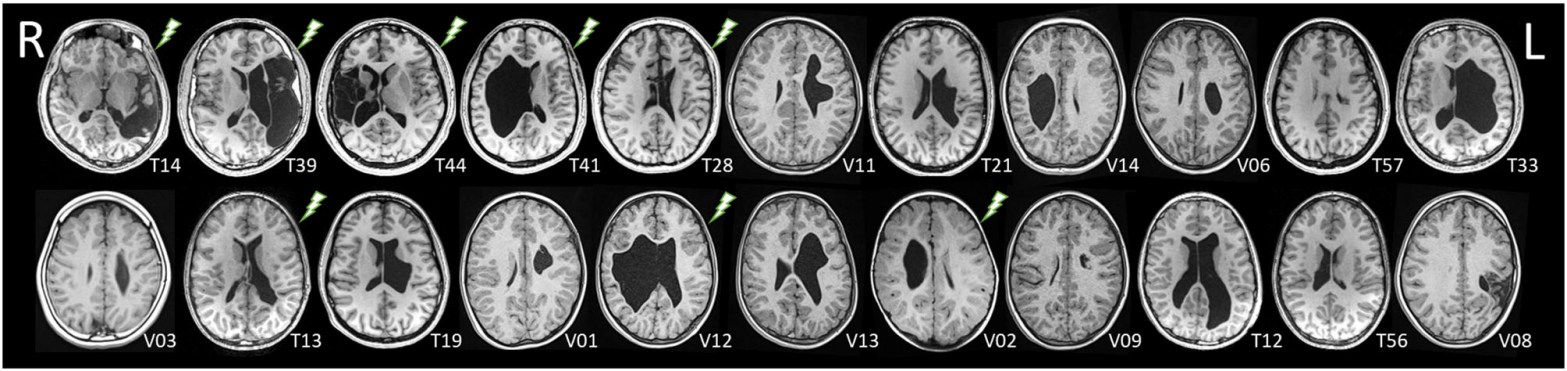
Axial slices of the T1-weighted datasets of the 22 patients with MRI data available, sorted by TONI scores (as in Table 1). No MRI was available in patient V04 due to dental braces. A “lightning” symbol marks patients with epilepsies. Radiological orientation, R, right; L, left.

Seven patients had right-sided lesions, 16 patients had left-sided lesions (Figure 2). In the 16 patients with left-sided lesions, functional MRI revealed left-lateralized language in 7/16 patients and right-lateralized language in 8/16 patients. Patient V09 could not be classified due to technical failure, and no patient showed bilateral language. Language lateralization indices ranged from +0.85 to −0.87 and correlated significantly with lesion size (*r* = −0.67, *p* < 0.05; Spearman rank): the larger the left-sided lesion, the stronger the right-sided language dominance.

In our healthy control group, the median in non-verbal intelligence (IQ = 103) was slightly above the population-based average (IQ = 100), but they did not differ from the population-based estimated distribution (Chi Square = 5.590; *p* = 0.232). Overperformance of our healthy peer group can therefore be ruled out as the decisive factor.

## Discussion

The major finding of our study was that epilepsy is a key risk factor for impaired non-verbal cognitive abilities in children with perinatal stroke. This has already been known for severe pharmaco-refractory epilepsies (3, 4, 21, 22); in the current study, we could demonstrate that negative effects can also be seen even in patients with well-controlled epilepsies, i.e., with seizure-freedom for at least 6 months. Despite our comprehensive assessment, none of the other potentially modifying factors lesion size, lesion side, language lateralization, or hand motor impairment played any role beyond the deleterious effects of epilepsy.

According to our analyses, **patients without epilepsies** after perinatal stroke can be expected to develop normal non-verbal cognitive functions. This is in line with other studies (3, 4) reporting non-verbal cognitive performances in the range of typically developing children for non-epileptic patients after perinatal stroke. Furthermore, we found no correlation between TONI scores and lesion size in this group (*r* = 0.02). This confirms the report by Lõo et al., demonstrating that, in non-epileptic children with PVI, lesion size did not correlate with cognitive abilities (7). These patients provide impressive examples of the neuroplastic potential of the developing human brain: Even patients with very large lesions (e.g., patient T33) have the potential to develop non-verbal intelligence in the normal range (TONI = 97). This neuroplastic potential basically of one hemisphere seems sufficient even in patients who shift language to the right hemisphere as a consequence of large left-sided lesions—thus apparently contradicting the “crowding hypothesis” (see below).

In contrast, **in patients with epilepsies**, we observed a significant impairment of non-verbal cognitive abilities, with scores ranging between 77 and 104, hence from “poor” to “average” cognitive ability according to the TONI-4-manual (28).

This is all the more striking as we had already excluded patients with pharmaco-refractory epilepsies (i.e., with ongoing seizures) and patients with a previous diagnosis of intellectual disability. Apparently, in the presence of epilepsy, the neuroplastic potential of the contra-lesional hemisphere is not sufficient to allow for an undisturbed cognitive development. Network formation must be expected to be an important step in establishing the neural substrates for higher cognitive functions. Several mechanism have been discussed how perinatal lesions lead to epileptogenesis later in life; it seems likely that epilepsy impairs neuroplasticity by interfering with network formation (42).

In order to exclude that our results are mostly driven by the one patient with seizure-freedom for <1 year (T13), we conducted all analyses again without this patient T13. We found that neither the results in the correlation analysis, nor the group comparisons changed after the exclusion of this patient; patients with epilepsy still scored significantly lower than controls (*p* = 0.007).

The same holds true for the multiple regression analysis; only the factor epilepsy (*R*^2^ = 0.20, beta = −0.444, *t* = −2.1, *p* < 0.05, one-tailed) was retained in the model as a statistically significant predictor, with epilepsy explaining 20 % of the variance in non-verbal intelligence.

In our cohort, none of the parameters characterizing epilepsy (i.e., epilepsy onset, time since last seizure, number of different antiepileptic drugs ever used, time between first and last seizure, or history of status epilepticus) correlated with non-verbal intelligence. This negative result must be interpreted, however, in the context of our small number of only eight patients with epilepsy and the limited dataset we obtained. We would expect that a more detailed analysis of a larger cohort of patients with stroke-induced epilepsy reveals such correlations.

Supporting our findings, impaired development of cognitive abilities in patients with epilepsies after perinatal stroke was also reported in previous studies (3, 4, 7). Two of these studies (3, 4) added a longitudinal aspect to this negative influence of epilepsy, describing a decline of cognitive functions over time associated with post-neonatal epilepsy in cohorts of children with perinatal AIS. Since our study was cross-sectional in nature, we are unable to provide such insights into the time-course of the development of cognitive impairment in our patients. Furthermore, these studies (3, 4, 7), reported on cohorts of patients with various seizure severities, ranging from seizure-free patients on anti-convulsive medication (as in most of our patients) to patients with drug-resistant epilepsies with ongoing seizures. The new aspect of our study in this respect, with seizure-freedom for at least 6 months as an inclusion criterion, was that the presence of even well-controlled epilepsy can significantly impair the development of non-verbal cognitive functions.

Nevertheless, children with **larger lesions** seem to be at higher risk to show impaired non-verbal cognitive abilities, since lesion size tended to correlate with non-verbal intelligence (*r* = −0.32). Our multiple regression analysis, however, demonstrated that this correlation is not an effect of lesion size *per se*, but arises as a consequence of the higher likelihood of larger lesions to result in epilepsies (*r* = 0.55). Hence, the effect of lesion size is biased by the higher likelihood of epilepsy in these patients.

In addition to the fact that motor impairment can artificially influence the test procedure itself, impaired motor abilities might limit a child's abilities to explore its environment, and thereby impair also the development of cognitive functions (29). Therefore, motor function is also an influencing factor when investigating cognitive outcome in children with unilateral perinatal stroke.

Similar to the lesion size analysis, children with hand motor impairment tend to show more severely impaired non-verbal cognitive abilities (*r* = −0.36). After inclusion of epilepsy as the first regressor, however, motor impairment ended up showing no additional significant effect in the regression model. This might indicate that epilepsy and motor impairment are epiphenomena of the same underlying feature of the lesion.

An interesting discrepancy arose when comparing our findings with previous data from our own group (14) regarding **the crowding hypothesis**. In this previous study (14), the degree of right-hemispheric language involvement did correlate with non-verbal cognitive parameters in a similar group of patients with unilateral PVI without epilepsies. Such a correlation was not only observed for the (potentially motor-contaminated) performance IQ scores of the Wechsler Intelligence Scales, but also for motor-free tests of visuospatial memory (Block-Tapping Test) and spatial ability (Tube Figures Test). To ensure that the presence of epilepsy in the current study did not wash out the influence of language lateralization or lesion size on the cognitive performances, we conducted the same analysis confined to patients without epilepsies—and again found no significant correlation between non-verbal intelligence and language lateralization (*n* = 12, *r* = −0.54, *p* = 0.09) or lesion size (*n* = 14, *r* = 0.02, *p* = 0.96).

This discrepancy may indicate that not all non-verbal cognitive functions are compromised to the same extent: In the current study, we found no evidence for non-verbal reasoning (as measured with the TONI) to be impaired by a right-shift of language. This is compatible with previous suggestions of spatial ability and reasoning tests being separable dimensions (43). Therefore, these two studies from our group seem to indicate that language shift to the right hemisphere may compromise some originary right hemispheric functions [such as visuospatial memory and spatial ability (14)] more than others (non-verbal reasoning—the current study).

Interestingly, the factor “**lesion side**” was irrelevant, which is compatible with recent studies that also failed to find such a difference using subtests of the Wechsler Intelligence Tests to assess non-verbal cognitive abilities (3, 4). This corroborates the hypothesis that cross-hemispheric reorganization may be an important mechanism underlying the neuroplastic potential of the developing human brain (44). In contrast, two studies reported lesion side-specific problems in cognition after perinatal strokes: In the first study (12), patients with perinatally acquired right-sided lesions were impaired in configural processing and made more global errors in reproducing memorized objects in drawing tasks, whereas patients with left-sided lesions were impaired in featural progressing and made more local errors. A possible explanation for this discrepancy is, again, that side-specific problems may exist for visuospatial memory functions, but that this side-specificity can not necessarily be transferred to the context of non-verbal reasoning as measured with the TONI in the current study. Second, in an earlier study from the same group (13), right-sided lesions lead to impaired visuospatial functions in pre-school children with perinatally acquired lesions. This specific impairment (which was revealed in drawing tasks) improved until school age, most likely by developing compensatory strategies. Therefore, the discrepancy to our data may not only be explained by the different cognitive functions tested, but also by the fact that we included only patients aged 8 years or older.

**Limitations** of our study include the following: First, we did not include patients with IQ <70. Therefore, we have certainly underestimated the impairment of cognitive functions caused by epilepsies. Second, we have not collected data on learning disabilities. Hence, we cannot exclude that despite average score in non-verbal cognitive abilities, our patients show deficits in these aspects of cognition. Third, we did not analyze EEG data of our patients. Therefore, we could not assess the potential influence of interictal epileptic activity on non-verbal cognitive development.

Fourth, our patients showed a wide age range. We controlled for this issue by using age-adjusted norms and controls, but the inhomogeneity of the group might still be a confounding factor. Fifth, our sample size is relatively small. Given the stringent inclusion criteria and the rarity of the underlying medical conditions, however, we attained an exceptional group size and used appropriate statistical approaches. We therefore interpret our results as valid. Sixth, our manual approach to determine lesion size is precise, but not at all topographically specific. We therefore cannot exclude that certain lesion topographies exert a more prominent effect on non-verbal cognitive abilities than what we report here. To this effect, lesion-symptom mapping approaches would have been necessary.

Seventh, AIS and PVI are two different pathological entities with different etiologies and different outcomes. Due to the small number of only 4 patients with AIS in our sample, we were unable to search for potential effects of lesion type.

Eighth, we did not control for the educational level of the parents—a factor which might also play a role in cognitive outcomes.

Ninth, with only two patients without CP in our cohort, we were unable to analyze the effect of presence vs. absence of CP on brain organization. Future studies could address this point by specifically recruiting patients without any motor impairment.

Our study has important **implications for patient counseling**: Patients with perinatal strokes without epilepsy can be expected to develop non-verbal intelligence within the range of typically developing children. If not hampered by epilepsy, the neuroplastic potential of one healthy hemisphere is able to support the development of normal non-verbal cognitive abilities, regardless of lesion size, lesion side, or language lateralization. Epilepsy, even when well-controlled, seems to substantially reduce this neuroplastic potential, resulting in impaired non-verbal abilities.

## Data Availability Statement

The original contributions presented in the study are included in the article/supplementary material, further inquiries can be directed to the corresponding author.

## Ethics Statement

The studies involving human participants were reviewed and approved by Ethik-Kommission an der Medizinischen Fakultät der Eberhard Karls Universität und am Universitätsklinikum Tübingen (Nr. 693/2014B01). Written informed consent to participate in this study was provided by the participants' legal guardian/next of kin.

## Author Contributions

KL and MS conceptualized and initiated the study. AG, MH, LS, and MS collected the data. PH, MW, LS, AG, and MS conceptualized and interpreted the (f)MRI and lesion size data. AG and MS analyzed and interpreted the data and prepared the manuscript. KL, MH, LS, MW, and PH reviewed the manuscript. All authors approved the final manuscript as submitted.

## Conflict of Interest

The authors declare that the research was conducted in the absence of any commercial or financial relationships that could be construed as a potential conflict of interest.
